# Diseases Admitted under the Care of the Physicians of the Westminster Hospital, from the 25th of June to the 24th of August, 1801

**Published:** 1801-09

**Authors:** 


					?t)8
Observations on the State of Diseases in London.
Difeafes admitted under the Care. of the Phyficians of the Wtjlminjler*
Hospital, from the 2$tb of June to the z\th of August, 1801.
Continued Fever - - - 24
Pleuritis ------ 2
Enteritis ------ 2
Eryfipelas ----- 1
Amenorrhcea - - - - 2
Anasarca ------ 8
Afcites 2
Aflhenia ------ 1
Afthma ------ j
Catarrh j
Cephalasa _____ j
Cholera *
Colic * 3
Diarrhoea ^
Dyfpepfia - - - - - 2
Dyfpnce'a ----- 5
Dyfuria
Dyfuria - - - - - - 3
Enterodynia - - - - 5
Galtrodynia ----- .3
Hsmoptos ----- 1
Hydrocephalus - - - - 1
Hyfteria ------ 2
Icterus ------ 1
Impetigo ----- 2
Leucorrhcea * - - - - J
Lithiafis ----- r I
Lumbago ----- 2
Menorrhagia - - - - 3
Obftipatio - - - - x
Paralyfis - - - - - ? .3
Pertuffis - - - - 1
Pleurodyne - - - - - 1
Rheumatifm - - - 10
Struma - i '. . _ ??
Syphilis - - x
Tabes j
Tulfis - _ _ _ Ja
Vertigo - - ^
Vomitus - r ?. ..-L &

				

